# Efficacy and Safety of Tetrahydrocurcuminoids for the Treatment of Canker Sore and Gingivitis

**DOI:** 10.1155/2020/6611877

**Published:** 2020-12-16

**Authors:** Muhammed Majeed, Shaheen Majeed, Kalyanam Nagabhushanam

**Affiliations:** ^1^Sami-Sabinsa Group Ltd., Peenya Industrial Area, Bangalore, Karnataka, India; ^2^Sabinsa Corporation, Payson, UT, USA; ^3^Sabinsa Corporation, East Windsor, NJ, USA

## Abstract

**Background:**

Tetrahydrocurcuminoids (THCs) are among the major metabolites of curcuminoids with a higher bioavailability and physiological stability and exhibit a broad spectrum of therapeutic activities. The objective of this study was to evaluate the efficacy of THCs in patients suffering from canker sore and gingivitis designed as an exploratory clinical trial.

**Methods:**

This is an open label prospective pilot clinical trial carried out at two clinical centers: Noble Hospital, Pune, Maharashtra, and Sri Venkateshwara Hospital, Bangalore, Karnataka in India. Participants were assigned to 21 days of treatment with chewable oral THCs supplement. Patients were instructed to self-administer one chewable tablet containing 100 mg of THCs twice daily for up to 21 days. This clinical trial was registered at a public Clinical Trial Registry in India (http://www.ctri.nic.in). Thirty-one canker sore and twenty-nine gingivitis patients participated in this study. Body mass index, throat numbness/relief, Visual Analog Scale (VAS) pain score, canker sore lesions, gingival appearance, inflammation and bleeding were assessed before and after treatment, at 14 and 21 days. Vital signs and laboratory parameters were assessed for safety.

**Results:**

THCs treatment significantly reduced the reddening at the site, difficulty in chewing, swallowing, and VAS pain score in the canker sore patients. Further, both single and multiple lesions were completely healed. In gingivitis patients, gingival appearance, bleeding, and inflammation were significantly reduced. No adverse effects were observed during the study.

**Conclusion:**

Overall, the findings of this study show that supplementation of THCs for 21 days reduced the pain and prevented the progression of the disease in patients suffering from canker sore and gingivitis without adverse side effects.

## 1. Introduction

The recurrent aphthous stomatitis (RAS), commonly known as canker sore or aphthous ulcer (aphthae), affects approximately 20% of the general population in North America [1]. Gingivitis, generally referred to as gum disease is associated with bleeding that can lead to periodontal disease, which may destroy gum and bone, resulting in loss of tooth if left untreated [2]. Microbial plaque and persistent inflammation in periodontal pockets are significant risk factors for gingivitis and periodontal diseases [3]. Currently, chlorhexidine and metronidazole are being used as an adjunct to mechanical cleaning. However, the above treatments are likely to have adverse side effects.

There is an increasing interest in using natural agents as dietary supplements for the prevention or treatment of oral diseases [4, 5]. Several plant products used in the ayurvedic practice are being reevaluated to determine their efficacy as an alternative therapy for the treatment of oral diseases and to alleviate the side effects of current drug regimens [6, 7]. Curcumin, the principal curcuminoid of turmeric from the rhizome of *Curcuma longa*, a member of the ginger family Zingiberaceae, is known for its antioxidant, anti-inflammatory, antibacterial, antitumor, and analgesic properties. Curcumin was also shown to have potential benefits in relieving pain and healing patients with aphthous ulcers [8, 9] and gingivitis [10–12].

**Figure 1:**
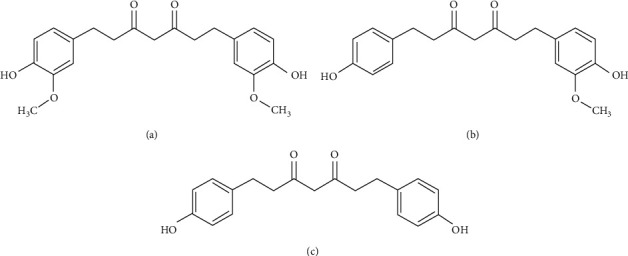
Structures of tetrahydrocurcuminoids (all the THCs exist as a mixture of keto and enol forms. Only the keto form is shown here). (a) THC. (b) THDMC. (c) THBDMC.

Tetrahydrocurcuminoids (THCs), the natural antioxidants, identified as the major metabolites of curcuminoids, consist of tetrahydrocurcumin (THC), tetrahydrodemethoxycurcumin (THDMC), and tetrahydrobisdemethoxycurcumin (THBDMC) (Figure 1), exhibiting similar physiological and pharmacological properties of curcumin [13].

Among the abovementioned three metabolites, THC is the major active one when curcuminoids are administered intraperitoneally [14] or orally as a dietary component to mice [15–19] and humans [20].

Tetrahydrocurcuminoids have also been isolated as a natural product from *Zingiber officinale* [21] and *Curcuma* species, *Curcuma zedoaria* [22], and *Curcuma longa* [23, 24]. Tetrahydrocurcumin is nearly colorless and lacks the double bonds, despite THC and curcumin possess similar *β*-diketone structures and phenolic groups [16]. Interestingly, the absence of a conjugated double bond in the hydrocarbon chain enables THCs to bind more efficiently with phospholipase A2 than curcumin [25]. Most importantly, THCs reported to have higher bioavailability and physiological stability than curcumin and are easily absorbed through the gastrointestinal tract [16]. Tetrahydrocurcuminoids possess an array of pharmacological properties, such as antioxidant, anti-inflammatory, antimicrobial, antidiabetic, anticancer, and antiallergic, as well as hepatoprotective, cardioprotective, neuroprotective, and nephroprotective activities surpassing curcuminoids in some of these activities [26]. Antioxidant supplements with their anti-inflammatory properties may play a potential role in the prevention and successful treatment of disorders associated with gingival tissues [27]. Several studies have demonstrated that THC is a potent antioxidant [16–19] and recent studies reinforce the earlier findings [28]. Experimental evidence from animal studies shows that THCs are more potent antioxidants than curcumin [16, 17, 28] and exert pronounced anti-inflammatory [28–30], antimicrobial [31], and anticancer activities [32].

Tetrahydrocurcuminoids are more potent than curcumin in preventing gingival microvascular dysfunction in diabetic rats by reducing oxidative stress [33]. Recently, Chhaparwal et al. [34] evaluated the efficacy and safety of THCs over 12 weeks in the treatment of oral leukoplakia showing that THCs (2%) when topically applied in a gel form was remarkably effective in alleviating clinical symptoms, suggesting that THCs could safely provide symptomatic relief for patients with oral leukoplakia. A recent 90-day subchronic and reproductive/developmental toxicity study demonstrated that repeated administration of 400 mg/kg THCs in rats was safe and was considered as the no observed adverse effect level (NOAEL) [35].

This open label prospective clinical pilot study, for the first time, was designed to investigate the safety and efficacy of THCs in patients suffering from canker sore and gingivitis. While randomized controlled trials are considered to be the standard approach for testing the safety and efficacy of drugs, a “prospective study” of patients with chronic diseases with a well-known baseline is often suggested in the complementary and alternative medicine field. A good outcome from such prospective clinical pilot studies could provide strong preliminary data on the safety and efficacy of THCs and will form the basis for future large-scale clinical trials.

## 2. Materials and Methods

### 2.1. Study Supplement

The study supplement, C3 Reduct® ODN (batch no. FD/JJ0816/C-03), containing 100 mg of 95% w/w naturally derived THCs from the rhizomes of *Curcuma longa* formulated as chewable tablets for oral dosage was provided by Sami Labs Limited, Bangalore, India.

### 2.2. Ethical Approval and Consent

This clinical trial was carried out at two centers: Noble Hospital, Pune, Maharashtra, and Sri Venkateshwara Hospital, Bangalore, Karnataka in India, from November 2016 to March 2017. The study was conducted with the full approval of the respective Institutional Ethics Committees in agreement with the International Conference on Harmonization guidelines on Good Clinical Practice and Helsinki Declaration. This clinical trial (CTRI/2017/01/007670) was registered at a public Clinical Trial Registry in India (http://www.ctri.nic.in). No amendments were made to the methods or planned endpoints after the initiation of the study. Written informed consent was obtained from all the participants of this trial.

### 2.3. Subjects

A total number of 60, both male and female subjects, between the age group of 18 to 60 years, were recruited at the study sites following the identical inclusion and exclusion criteria (Supplementary Table 1). Based on the selection criteria, 31 patients having mouth ulcer (canker sore) with throat pain and 29 patients with mild to moderate gingivitis were enrolled in this study. Among the 31 subjects enrolled in the canker sore group, 8 were females and 23 were males. In the gingivitis group, out of the 29 subjects, 3 were females and 26 were males. All the subjects enrolled had no other health issues.

### 2.4. Study Design and Interventions

**Figure 2:**
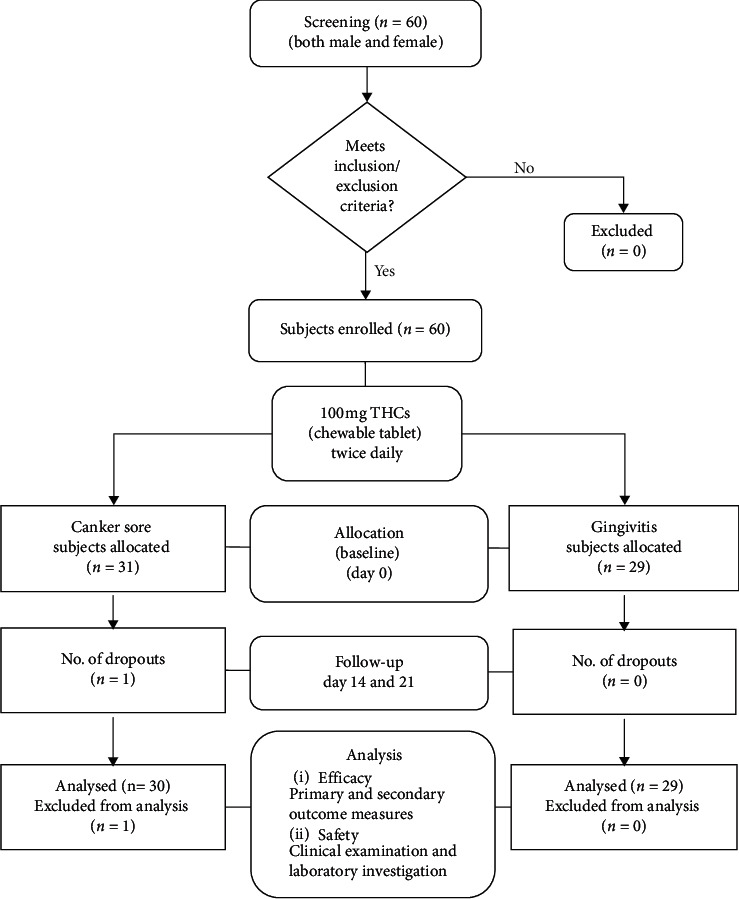
Study design flow chart on subject participation, efficacy, and safety assessment.

The enrolled subjects were screened on day 0 (baseline) and assigned to the respective canker sore and gingivitis groups for treatment were instructed to self-administer one chewable tablet containing 100 mg of THCs twice daily for up to 21 days. The subjects were allowed to continue on their regular diet. The efficacy was evaluated on days 14 and 21 during the treatment period. Out of the 31 subjects in the canker sore group, one female subject dropped out after 14 days and the remaining 30 subjects completed the study. In the gingivitis group, all 29 subjects completed the study (Figure 2).

### 2.5. Outcome Measures

The body mass index (BMI) was documented, before (baseline) and after 14 and 21 days of treatment. The number of ulcers healed, size reduction, and duration for the complete healing were assessed for efficacy evaluation. In addition, the VAS (Visual Analog Scale) score for pain reduction was also assessed to determine the efficacy. Further, the VAS pain scores of 0 (no pain), 1 (mild), 2 (moderate), and 3 (severe) were used for the assessment of pain intensity in canker sore patients. Clinical photographs of the lesion sites were captured to assess the treatment efficacy. Similarly, for gingivitis, Löe and Silness gingival index and subjective and objective criteria were assessed.

### 2.6. Safety Assessments

Safety assessments performed included clinical examination of vital signs, as well as laboratory/hematological investigations. Blood samples were collected from subjects for the analysis of hematological and laboratory parameters. Adverse events and concomitant medication, if any, were reviewed during the study period.

### 2.7. Statistical Methods

The data gathered were sorted, tabulated, and subjected to appropriate statistical analysis using SAS 9.1.3 software package (Cary, NC, USA). For statistical purposes, the screening scores were considered as baseline. Results are expressed as mean ± SD or median scores. The baseline and THCs treatment data generated were compared at the two-sided 5% significance level. Paired *t*-tests were performed to assess the changes in the safety parameters. A comparative analysis on the efficacy was further evaluated using additional statistical methods, such as Cochran's *Q* test, McNemar test, Friedman ANOVA, and Wilcoxon signed-rank test. For all the statistical tests, *p* < 0.05 was considered significant.

## 3. Results

### 3.1. Patient Population

The gender distribution in the canker sore group was 23 (74%) males and 8 (26%) females while 26 (90%) males and 3 (10%) females were in the gingivitis group. The data analysis performed in the canker sore group excluding the 1 subject who was dropped out of the study did not influence the overall interpretation or conclusion of the findings.

### 3.2. Effect of THCs on Clinical Parameters

Physical examination after treatment, including the general appearance of the subjects, vital signs of the heart, lungs, abdomen, extremities, and neurological functions, was found to be normal in all the subjects. In addition, a comparative evaluation of clinical parameters, including systolic and diastolic blood pressure (BP), pulse rate, heart rate, respiratory rate, and BMI after treatment, was also found to be in the normal range for all the patients in both canker sore and gingivitis study groups (Supplementary Figures 1 and 2). Similarly, a comparison of clinical parameters performed for both canker sore and gingivitis groups using paired *t*-test showed no significant difference between baseline and after treatment, *p* > 0.05). Overall assessment of the clinical parameters revealed that all the subjects were in the normal range and were not altered by treatment with THCs.

### 3.3. Effect of THCs on Canker Sore Symptoms

The effect of THCs on the clinical signs and symptoms of canker sore patients are provided in Table 1.

The analysis showed a reduction in pain, burning, and reddening after 14 days of treatment indicating a minimum of approximately 50% decrease in the above symptoms. Similarly, difficulty in chewing and swallowing was reduced. At the end of the 21 days of treatment, a large reduction in the pain score, burning, reddening, difficulty in chewing, *and* swallowing in patients was observed. A statistical comparison of the clinical signs and symptoms using the Cochran's *Q* test also showed a significant reduction in pain, burning, reddening, and difficulty in chewing and swallowing (*p* < 0.001). No change in the bleeding score observed at the end of the treatment (*p*=0.135) indicated further deterioration of the gums.

**Figure 3:**
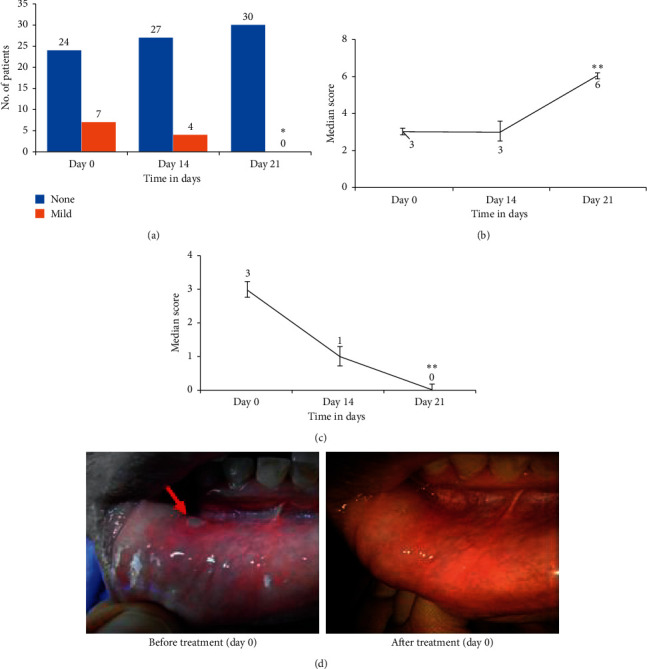
Comparative analysis of data on clinical signs and symptoms of canker sore patients before (day 0) and after treatment (days 14 and 21) with THCs. (a) No. of patients with throat numbness at different time points of treatment, ^*∗*^*p* < 0.05 based on McNemar and Cochran's *Q* tests and as compared by chi-square test. (b) Median throat relief score at three points of treatment determined using Friedman ANOVA, ^*∗∗*^*p* < 0.001. (c) Median VAS pain score at different time points of treatment determined using Friedman ANOVA, ^*∗∗*^*p* < 0.001. (d) Visual evaluation.

Further, the Cochran's *Q* test showed that the 7 subjects who complained about mild numbness at screening were completely relieved after treatment (*p*=0.025) (Figure 3(a) and Supplementary Table 2).

Similarly, the McNemar test performed also supported the absence of throat numbness in all the subjects after treatment (*p* < 0.05). A comparison of throat relief score using the Wilcoxon signed-rank test showed a significant improvement after treatment (*p* < 0.001) (Supplementary Table 2), which is further supported by Freedman ANOVA, *p* < 0.001 (Figure 3(b)).

A progressive reduction in the VAS pain score was noticeable even after 14 days of treatment. A highly significant decrease in the VAS scores was observed after 21 days. In addition, the VAS score analyzed using the Freedman ANOVA test showed that, out of the 30 subjects, a median VAS score of 3, indicating the high end of the pain scale, was recorded in majority of the subjects before treatment. The VAS score was significantly reduced to 0 after THCs treatment implying no pain in 93.3% of the subjects (*p* < 0.001) (Figure 3(c) and Supplementary Table 2).

Pairwise comparison using the Wilcoxon signed-rank test suggested a statistically significant difference between all the paired treatment time points (*p* < 0.05). Overall, the abovementioned findings reveal that THCs treatment significantly alleviated the symptoms in canker sore patients.

### 3.4. Effect of THCs on Canker Sore Lesions

After 21 days of treatment with THCs, the canker sore lesions were significantly reduced in all the patients. Before treatment, a majority of 26 patients had a single lesion with a size ranging between 1 and 4 mm, while 5 had more than one lesion in the same size range. After 14 days of treatment, the number of subjects with multiple lesions decreased slightly from 5 (16%) to 4 (12.9%). Out of the 31 subjects enrolled, one subject dropped out after 14 days and the remaining 30 subjects completed the study. At the end of the study, all the 30 (100%) patients with both the single and multiple lesions were completely healed. Further, the photographic evaluation confirmed the clinical observations on the significant reduction of canker sore lesions (Figure 3(d)).

### 3.5. Effect of THCs on Gingivitis

**Figure 4:**
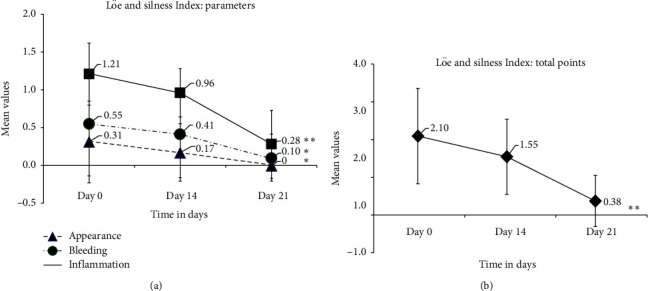
Effect of THCs on gingivitis. (a) Line plots showing differences in Löe and Silness Plaque Indices (PI) (mean ± SD) of gingivitis patients such as appearance, bleeding, and inflammation before (day 0) and after treatment (days 14 and 21) with THCs analyzed using the Friedman ANOVA. (b) Total points (mean ± SD) before (day 0) and after treatment (days 14 and 21) with THCs analyzed using the Wilcoxon signed-rank test. ^*∗∗*^*p* < 0.001 for gingival (gum) appearance and bleeding; ^*∗∗*^*p* < 0.001 for inflammation and total points.

After treatment with THCs, the burning sensation was completely absent in the majority of the patients that was further confirmed by McNemar statistical analysis (*p* < 0.05). The staining of teeth was also reduced after treatment. Improvements in the gingivitis symptoms, such as dryness/soreness and ulcer formation were observed after 14 days and these improvements were effective until the end of the treatment period (Table 2). Improvement in the gingival index score was observed after 14 days of treatment. The mean gingival index scores were significantly reduced appearance from 0.31 ± 0.54 to 0.17 ± 0.38, bleeding from 0.55 ± 0.69 to 0.41 ± 0.57, and gingival inflammation from 1.21 ± 0.41 to 0.96 ± 0.32. Overall, the mean of the total gingival index point score was also reduced from 2.10 ± 1.26 (baseline) to 1.55 ± 0.95 with a median of 1 after treatment (Figures 4(a) and 4(b)).

Similarly, at the end of 21 days of treatment, a significant improvement in the gingival index score was observed compared to the baseline in all the patients. The mean gingival scores were further reduced, appearance to 0, bleeding to 0.10 ± 0.31, and gingival inflammation to 0.28 ± 0.45, from the baseline values. The mean total gingival score was also reduced to 0.38 ± 0.68 with a median of 0 from the baseline value. The Löe and Silness index score evaluated using the Friedman ANOVA test showed that the gingivitis appearance and bleeding scores were significantly reduced after treatment (*p* < 0.01) (Figure 4(a)). A highly significant reduction in gingival inflammation and the total gingival points score was noticed after treatment (*p* < 0.001).

A comparative analysis of the efficacy of THCs performed using the Wilcoxon signed-rank test revealed a highly significant reduction in the gingival index score for appearance (*p*=0.008), bleeding (*p*=0.002), gingival inflammation (*p* < 0.001), and the total point of all parameters (*p* < 0.001) (Supplementary Table 3). Overall, after the treatment, a substantial reduction in gingivitis plaque was observed in all 29 patients.

### 3.6. Safety Evaluation of THCs

There were no significant changes observed in any of the vital signs, as well as clinical and hematological parameters in patients with both canker sore and gingivitis during the study period. No adverse events were reported. Overall, the results of this study demonstrated that THCs treatment was completely safe and acceptable as an oral supplement (Supplementary Figures 1 and 2; Supplementary Tables 4 and 5).

## 4. Discussion

Earlier studies have shown that THC can protect the skin matrix by minimizing inflammation and by improving skin elasticity and tightness, suggesting that THC can be used as a safe agent for wound healing [36]. The present clinical trial, for the first time, demonstrated that oral supplementation of THCs alleviated the symptoms within 21 days of treatment in patients with canker sore and gingivitis and effectively prevented further progression of the disease without adverse effects. Curcumin-based mouthwash was shown to reduce gingival inflammation and plaque in patients with chronic gingivitis [10, 37, 38]. Previous studies also showed that turmeric mouthwash containing curcumin significantly reduced the total microbial count [39]. Arguably, THCs derived from the curcuminoids with a higher bioavailability and physiological stability and with more potent antioxidant [16–19, 28], anti-inflammatory [28–30], antimicrobial [31], and anticancer activity [32] than curcumin could be an effective agent for protecting oral health.

In addition to the anti-inflammatory activities of THCs, a vast number of *in vitro* and *in vivo* studies have shown that THCs exert higher antioxidant activity than curcumin [16, 17, 19, 40]. THCs have been shown to interfere with free radicals effectively to reduce oxidative stress and thus prevent adverse chain reactions leading to inflammation [41]. San Miguel et al. [42] demonstrated that THCs, a natural antioxidant, in combination with resveratrol and phloretin, regulate fibroblast migration and proliferation during gingival healing or periodontal repair.

Similarly, several other studies have acknowledged that THCs exhibit potent anti-inflammatory activity, inhibiting LPS-induced production of NO, expression of iNOS and COX-2, proinflammatory cytokines TNF-*α* and IL-6, and activation of NF-*κ*B p65 [28, 30, 43] with a stronger safety profile than curcumin where the production of proinflammatory mediators has been reduced and thus ameliorating inflammation [29]. Further, Singh and Jain [44] reported that THC showed significant antimicrobial activity when compared with standard drugs such as kanamycin and fluconazole for antibacterial and antifungal activities, respectively.

Regarding the clinical efficacy, the overall findings from this study revealed that THCs effectively inhibited burning and reddening, reduced throat numbness, and relieved throat pain with the disappearance of canker sore lesions. No change in the bleeding was observed after treatment when compared with the baseline indicating no further deterioration of the gums. Moreover, the photographic evaluation before and after treatment showed the absence of canker sore lesions after treatment with THCs.

Earlier, Promrug [33] showed that supplementation of THCs reduced MDA-induced oxidative stress and prevented leukocyte adhesion improving the gingival blood flow and protecting the gingival microvascular function in diabetic rats. For the first time, it is shown that THCs supplementation significantly improved gingival appearances, with no bleeding or inflammation in patients suffering from gingivitis.

Earlier reports suggest that THCs are more stable in the plasma than curcumin [45]. Therefore, the efficacy of THCs in protecting oral health could also be attributed to its higher stability and absorption than curcumin [13, 19, 28–30]. Most importantly, safety assessments confirmed that THCs are safe and no adverse side effects were experienced by the patients during the study period. The overall findings from this study demonstrated that oral administration of THCs is safe and efficacious in patients suffering from canker sore and gingivitis.

The present study has some strengths and a few limitations. The limitations include relatively a small number of subjects and lack of a placebo group. However, this pilot clinical study provided strong preliminary data on the safety and efficacy of THCs against canker sore and gingivitis and will form the basis for future large-scale clinical trials.

## 5. Conclusion

Oral supplementation of THCs is shown to be an effective way of reducing inflammation and pain associated with gingivitis. The various gingival scores uniformly registered a reduction progressively over the course of the study period. A statistically significant gingival reduction was observed at the end of the study. In the assessment of efficacy of THC in patients with canker sore lesions, complete disappearance of the lesions was observed at the end of the 21-day study period. The clinical treatment was safe with no significant changes in the vital signs as well as clinical and hematological parameters in the subjects involved in the study. Thus, the oral treatment with THCs is an effective and safe way to reduce pain and suffering in subjects from gingivitis and recurrent aphthous stomatitis.

## Figures and Tables

**Table 1:** Clinical signs and symptoms before and after treatment with THCs in canker sore patients.

Signs and symptoms	Baseline		Treatment with THCs				Cochran's *Q* test	*p* value
	Day 0		Day 14		Day 21			
	*N*	*N*%	*N*	*N*%	*N*	*N*%		
Bleeding	2	6.5	0	0.0	0	0.0	4	0.135^a^
Pain at the site	23	74.2	13	41.9	2	6.7	30.1	<0.001^b^
Burning at the site	16	51.6	2	6.5	1	3.3	28.1	<0.001^b^
Reddening at the site	21	67.7	8	25.8	0	0.0	27.64	<0.001^b^
Inflammation at the site	0	0.0	0	0.0	0	0.0	—	—^c^
Swelling and pain at lymph node	0	0.0	0	0.0	0	0.0	—	—^c^
Difficulty in chewing	12	38.7	5	16.1	0	0.0	16.77	<0.001^b^
Difficulty in swallowing	18	58.1	6	19.4	1	3.3	22.9	<0.001^b^
Injury to mouth	1	3.2	—	—	—	—	—	—

*N* = patient count; *N*% = percentage of patient count. Cochran's *Q* test was performed for the comparison of signs and symptoms before and after treatment. ^a^THCs treatment prevented further deterioration of the gums, *p* > 0.05. ^b^Significant reduction after THCs treatment, *p* < 0.001. ^**c**^No statistical test was performed since no change was observed between baseline and treatment.

**Table 2:** Clinical signs and symptoms before and after treatment with THCs in gingivitis.

Signs and symptoms		Baseline		Treatment with THCs			
		Day 0		Day 14		Day 21	
		*N*	*N*%	*N*	*N*%	*N*	*N*%
Taste acceptability^a^	Acceptable	29	100.0	29	100.0	29	100.0
	Unacceptable	0	0.0	0	0.0	0	0.0

Burning sensation^b^	Absent	16	55.2	27	93.1	29	100.0
	Present	13	44.8	2	6.9	0	0.0

Dryness/soreness^c^	Absent	28	96.6	29	100.0	29	100.0
	Present	1	3.4	0	0.0	0	0.0

Ulcer formation^c^	Absent	27	93.1	29	100.0	29	100.0
	Present	2	6.9	0	0.0	0	0.0

Staining of teeth^c^	Absent	15	51.7	13	44.8	18	62.1
	Present	14	48.3	16	55.2	11	37.9

Staining of tongue^a^	Absent	28	96.6	29	100.0	28	96.5
	Present	1	3.4	0	0.0	1	3.5

Allergy^a^	Absent	29	100.0	29	100.0	29	100.0
	Present	0	0.0	0	0.0	0	0.0

*N* = patient count; *N*% = percentage of patient count. McNemar's statistical test was used for the comparison of various gingivitis criteria (signs and symptoms) before and after treatment. ^a^No statistical test was performed since the patient's response was constant between baseline and treatment. ^b^Burning sensation was significantly reduced in patients after treatment (days 14 and 21) compared to baseline, *p* < 0.05. ^c^No significant difference was observed between the baseline and THCs treatment, *p* > 0.05.

## Data Availability

The data used to support the findings of this study are available with the article.

## Supplementary Materials

Supplementary Materials Supplementary Figure 1: Line plots showing data of BMI and clinical parameters (mean ± SD) of canker sore patients before and after treatment with THCs (days 14 and 21). The repeated measure of the analysis of variance (ANOVA) of the means of the clinical parameters across the study period showed no significant difference, *p* > 0.05. Supplementary Figure 2: Line plots showing data of BMI and clinical parameters (mean ± SD) of gingivitis patients before and after treatment with THCs (days 14 and 21). The repeated measure of the analysis of variance (ANOVA) of the means of the clinical parameters across the study period showed no significant difference, *p* > 0.05. Supplementary Table 1: Summary of selection criteria. Supplementary Table 2: A comparative analysis of clinical symptoms in canker sore patients treated with THCs. Supplementary Table 3: A comparative analysis of Löe and Silness Index in gingivitis patients treated with THCs. Supplementary Table 4: Laboratory safety investigational parameters assessed before (baseline) and after THCs treatment in canker sore patients. Supplementary Table 5: Laboratory safety investigational parameters assessed before (baseline) and after THCs treatment in gingivitis patients.
