# Supplementary material for: Efficacy and Safety of Tetrahydrocurcuminoids for the Treatment of Canker Sore and Gingivitis
Source: Evid Based Complement Alternat Med. 2020 Dec 16;2020:6611877. doi: 10.1155/2020/6611877 (PMC7758131; doi:10.1155/2020/6611877)
Supplement: Supplementary Materials — Supplementary Figure 1: Line plots showing data of BMI and clinical parameters (mean ± SD) of canker sore patients before and after treatment with THCs (days 14 and 21). The repeated measure of the analysis of variance (ANOVA) of the means of the clinical parameters across the study period showed no significant difference, p > 0.05. Supplementary Figure 2: Line plots showing data of BMI and clinical parameters (mean ± SD) of gingivitis patients before and after treatment with THCs (days 14 and 21). The repeated measure of the analysis of variance (ANOVA) of the means of the clinical parameters across the study period showed no significant difference, p > 0.05. Supplementary Table 1: Summary of selection criteria. Supplementary Table 2: A comparative analysis of clinical symptoms in canker sore patients treated with THCs. Supplementary Table 3: A comparative analysis of Löe and Silness Index in gingivitis patients treated with THCs. Supplementary Table 4: Laboratory safety investigational parameters assessed before (baseline) and after THCs treatment in canker sore patients. Supplementary Table 5: Laboratory safety investigational parameters assessed before (baseline) and after THCs treatment in gingivitis patients. [file 6611877.f1.zip › 6611877.f1/eCAM_Majeed et al_THCs_Suppl. Figure 1.doc]

**Majeed et al.**

**Journal Title**: **Evidence-Based Complementary and Alternative Medicine *(*eCAM*)***

**Article Title: Efficacy and Safety of Tetrahydrocurcuminoids for the treatment of Canker Sore and Gingivitis**

**Supplementary Material**

**Legend**

**Supplementary Figure 1:** Line plots showing data of BMI and clinical parameters (mean±SD) of canker sore patients before (Day 0) and after treatment (days14 and 21) with THCs. The repeated measure of the analysis of variance (ANOVA) of the means of the clinical parameters across the study period showed no significant difference, p> 0.05.

**Supplementary Figure 1**
