# Supplementary material for: Efficacy and Safety of Tetrahydrocurcuminoids for the Treatment of Canker Sore and Gingivitis
Source: Evid Based Complement Alternat Med. 2020 Dec 16;2020:6611877. doi: 10.1155/2020/6611877 (PMC7758131; doi:10.1155/2020/6611877)
Supplement: Supplementary Materials — Supplementary Figure 1: Line plots showing data of BMI and clinical parameters (mean ± SD) of canker sore patients before and after treatment with THCs (days 14 and 21). The repeated measure of the analysis of variance (ANOVA) of the means of the clinical parameters across the study period showed no significant difference, p > 0.05. Supplementary Figure 2: Line plots showing data of BMI and clinical parameters (mean ± SD) of gingivitis patients before and after treatment with THCs (days 14 and 21). The repeated measure of the analysis of variance (ANOVA) of the means of the clinical parameters across the study period showed no significant difference, p > 0.05. Supplementary Table 1: Summary of selection criteria. Supplementary Table 2: A comparative analysis of clinical symptoms in canker sore patients treated with THCs. Supplementary Table 3: A comparative analysis of Löe and Silness Index in gingivitis patients treated with THCs. Supplementary Table 4: Laboratory safety investigational parameters assessed before (baseline) and after THCs treatment in canker sore patients. Supplementary Table 5: Laboratory safety investigational parameters assessed before (baseline) and after THCs treatment in gingivitis patients. [file 6611877.f1.zip › 6611877.f1/eCAM_Majeed et al_THCs_Suppl. Material_Tables1-5_Clean.doc]

**Majeed et al.**

**Journal Title**: **Evidence-Based Complementary and Alternative Medicine *(*eCAM*)***

**Article Title: Efficacy and Safety of Tetrahydrocurcuminoids for the treatment of Canker Sore and Gingivitis**

**Supplementary Material**

**Supplementary Table 1:** Summary of selection criteria

| **Inclusion criteria** |  | **Exclusion criteria** |
| --- | --- | --- |
| 1. Male and/or female – Healthy, with no systemic disease (diabetes) |  | 1. Smokers |
| 2. Age between 18 to 60 years |  | 2. Presence of any systemic hematological problems |
| 3. Patients having mouth ulcers with throat pain or patients with mild to moderate gingivitis |  | 3. Drug or alcohol abuse |
| 4. Patients having erupted teeth |  | 4. Under systemic or topical corticosteroid therapy |
| 5. Willing to come for regular follow-up visits |  | 5. Pregnant women or lactating mothers |
| 6. Able to give written informed consent |  | 6. History of HIV and other viral infections |
|  |  | 7. History of any antibiotic therapy in the past 3 months |
|  |  | 8. History of oral prophylaxis within 6 months previous to the study |
|  |  | 9. Patients with mouth breathing habit |
|  |  | a) Patients using orthodontic and prosthodontic appliances |
|  |  | b) Are on analgesics or antiseptics  10. Patients who have received any periodontal therapy in the past 6 months. |

## **Supplementary Table 2**: A comparative analysis of clinical symptoms in canker sore patients treated with THCs

| **Characteristics** | **Before and after Treatment with THCs** | | | | | | p-value |
| --- | --- | --- | --- | --- | --- | --- | --- |
|  | Day - 0 | | Day - 14 | Day 21 | | |  |
| Evaluable subjects (%) | 31 (100) | | 31 (100) | 30 (100) | | |  |
| **Throat numbness scale** [**No**. (%)] | | | | | | | |
| None | 24 (77.4%) | 27 (87.1%) | | | 30 (100%) | 0.025^‡^ | |
| Mild | 7 (22.6%) | 4 (12.9%) | | | 0 |  |  |
| **Throat relief scale** [**No**. %)] | | | | | | | |
| No relief (1) | 1 (3.2%) | 0 (0%) | | | 0 | < 0**.**001**^†^** | |
| Slight (2) | 4 (12.9%) | 13 (41.9%) | | | 0 |  |  |
| Mild (3) | 25 (80.6%) | 5 (16.1%) | | | 0 |  |  |
| Considerable (4) | 1 (3.2%) | 3 (9.7%) | | | 0 |  |  |
| Almost complete (5) | 0 | 8 (25.8%) | | | 8 (26.7%) |  |  |
| Complete (6) | 0 | 2 (6.5%) | | | 22 (73.3%) |  |  |
| Median relief score | 3 | 3 | | | 6 |  | |
| **Vas score** [**No**. %)] | | | | | | | |
| 0 | 0 | | 7 (22.6%) | 28 (93.3%) | | | < 0**.**001**^†^** |
| 1 | 2 (6.5%) | | 15 (48.4%) | 0 | | |  |
| 2 | 7 (22.6%) | | 8 (25.8%) | 2 (6.7%) | | |  |
| 3 | 21 (67.7%) | | 1 (3.2%) | 0 | | |  |
| 4 | 1 (3.2%) | | 0 | 0 | | |  |
| Median VAS score | 3 | | 1 | 0 | | |  |

Statistical analysis: ^‡^Using McNemar test; **†**Using Friedman ANOVA

**Supplementary Table 3**: A comparative analysis of Löe and Silness index in gingivitis patients treated with THCs

| **Löe & Silness index** | **Before Treatment** (**Day - 0**) | **After Treatment** (**Day - 21**) | **p-value**** |
| --- | --- | --- | --- |
|  | **Mean (Median) [SD]** | **Mean (Median)** |  |
| Appearance | 0.42 (0) [0.65] | 0.00 (0) [0] | 0.008 |
| Bleeding | 0.58 (0) [0.72] | 0.04 (0) [0.2] | 0.002 |
| Inflammation | 1.25 (1) [0.44] | 0.08 (0) [0.28] | < 0.001 |
| Total points | 2.25 (2) [1.33] | 0.13 (0) [0.45] | < 0.001 |

Statistical analysis: **Using Wilcoxon signed-rank test

**Supplementary Table 4:** Laboratory safety investigational parameters assessed before (baseline) and after THCs treatment in canker sore patients.

|  | **Baseline (day 0) vs. Day 21** | | | | | | |  | **Baseline (day 0) vs. Day 21^a^** | | | | | |
| --- | --- | --- | --- | --- | --- | --- | --- | --- | --- | --- | --- | --- | --- | --- |
|  | **Baseline (Screening, day 0)** | | |  | **Day 21** | | |  | **Mean Difference** | **Std. Deviation** | **95% Confidence Interval of the Difference** | | **t**- **value** | **p value** |
| **Parameter** | **Mean  (n=31)** | **Std. Deviation** | **Std. Error** |  | **Mean  (n=26)** | **Std. Deviation** | **Std. Error** |  |  |  |  |  |  |  |
|  |  |  |  |  |  |  |  |  |  |  | **Lower** | **Upper** |  |  |
| Hemoglobin (g/dL) | 14.69 | 1.77 | 0.32 |  | 15.08 | 1.81 | 0.36 |  | -0.43 | 1.51 | -1.04 | 0.18 | -1.44 | 0.16^b^ |
| Hematocrit (%) | 42.77 | 4.9 | 0.88 |  | 43.92 | 5.44 | 1.07 |  | -1.06 | 4.74 | -2.98 | 0.85 | -1.14 | 0.27^b^ |
| Erythrocyte count (10^6^/μL) | 5.04 | 0.5 | 0.09 |  | 6.52 | 7.69 | 1.51 |  | -1.46 | 7.53 | -4.51 | 1.58 | -0.99 | 0.33^b^ |
| Platelet Count (10^3^/μL) | 248064.52 | 49623.2 | 8912.59 |  | 258500 | 50261.91 | 9857.17 |  | -9384.62 | 58225.82 | -32902.53 | 14133.30 | -0.82 | 0.42^b^ |
| Leukocyte count (10^3^/μL) | 7761.29 | 1983.55 | 356.26 |  | 7773.08 | 2345.47 | 459.99 |  | -7.69 | 2880.13 | -1171.00 | 1155.62 | -0.01 | 0.99^b^ |
| Neutrophils (%) | 64.1 | 8.46 | 1.52 |  | 58.58 | 7.97 | 1.56 |  | - | - | - | - | - | -^c^ |
| Lymphocytes (%) | 27.23 | 8.56 | 1.54 |  | 29.73 | 10.11 | 1.98 |  | -1.92 | 11.74 | -6.66 | 2.82 | -0.84 | 0.41^b^ |
| Monocytes (%) | 4.26 | 5.42 | 0.97 |  | 2.85 | 1.16 | 0.23 |  | 1.73 | 5.77 | -0.60 | 4.06 | 1.53 | 0.14^b^ |
| Eosinophils (%) | 4.32 | 3.87 | 0.69 |  | 7 | 3.71 | 0.73 |  | - | - | - | - | - | -^c^ |
| Basophils (%) | 0 | 0 | 0 |  | 0.27 | 1.37 | 0.27 |  | -0.27 | 1.37 | -0.82 | 0.29 | -1.00 | 0.33^b^ |
| Mean cell volume (fL) | 84.9 | 5.01 | 0.9 |  | 86.74 | 7.49 | 1.47 |  | -1.94 | 7.09 | -4.80 | 0.93 | -1.39 | 0.18^b^ |
| Mean corpuscular hemoglobin (pg) | 29.16 | 2.12 | 0.38 |  | 30.13 | 2.71 | 0.53 |  | - | - | - | - | - | -^c^ |
| Mean corpuscular hemoglobin concentration (g/dL) | 34.33 | 1.11 | 0.2 |  | 34.75 | 1.39 | 0.27 |  | -0.57 | 1.52 | -1.18 | 0.04 | -1.92 | 0.07 ^b^ |

^a^Pair wise comparison of each parameter between baseline (day 0) and day 21 was performed using paired t-test.

^b^No significant difference was observed in most of the parameters between baseline (day 0) and day 21.

^c^ THCs modulated neutrophils, eosinophils and mean corpuscular hemoglobin parameters (p<0.05).

**Supplementary Table 5:** Laboratory safety investigational parameters assessed before (baseline) and after THCs treatment in gingivitis patients

|  | **Baseline (Day 0) and Day 21** | | | | | | |  | **Baseline (Day 0) vs. Day 21^a^** | | | | | | |
| --- | --- | --- | --- | --- | --- | --- | --- | --- | --- | --- | --- | --- | --- | --- | --- |
|  | **Baseline (Screening, day 0)** | | |  | **Day 21** | | |  | **Mean Difference** | **Std. Deviation** | **Std. Error Mean** | **95% Confidence Interval of the Difference** | | **t- value** | **p value** |
| **Parameter** | **Mean  (n=29)** | **Std. Deviation** | **Std. Error** |  | **Mean  (n=25)** | **Std. Deviation** | **Std. Error** |  |  |  |  |  |  |  |  |
|  |  |  |  |  |  |  |  |  |  |  |  | **Lower** | **Upper** |  |  |
| Hemoglobin (g/dL) | 15.22 | 1.49 | 0.28 |  | 15.04 | 1.3 | 0.26 |  | 0.22 | 1.19 | 0.24 | -0.27 | 0.71 | 0.923 | 0.365^b^ |
| Hematocrit (%) | 44.09 | 3.78 | 0.7 |  | 44.19 | 3.29 | 0.66 |  | 0.13 | 4.4 | 0.88 | -1.69 | 1.95 | 0.15 | 0.882^b^ |
| Erythrocyte count (10^6^/μL) | 4.95 | 0.6 | 0.11 |  | 6.32 | 7.59 | 1.52 |  | -1.42 | 7.74 | 1.55 | -4.62 | 1.77 | -0.92 | 0.367^b^ |
| Platelet Count (10^3^/μL) | 262551.72 | 64679.75 | 12010.73 |  | 273560 | 48872.35 | 9774.47 |  | -13440 | 63132.85 | 12626.57 | -39499.96 | 12619.96 | -1.064 | 0.298^b^ |
| Leukocyte count (10^3^/μL) | 7762.07 | 2125.76 | 394.74 |  | 8384 | 2490.93 | 498.19 |  | -632 | 3234.41 | 646.88 | -1967.1 | 703.1 | -0.977 | 0.338^b^ |
| Neutrophils (%) | 59.31 | 6.94 | 1.29 |  | 62.76 | 7.51 | 1.5 |  | -3.52 | 9.29 | 1.86 | -7.35 | 0.31 | -1.895 | 0.07^b^ |
| Lymphocytes (%) | 29.59 | 8.76 | 1.63 |  | 26.72 | 7.11 | 1.42 |  | 2.56 | 12.57 | 2.51 | -2.63 | 7.75 | 1.019 | 0.319^b^ |
| Monocytes (%) | 4.14 | 1.96 | 0.36 |  | 3.56 | 1.39 | 0.28 |  | 0.84 | 2.1 | 0.42 | -0.02 | 1.7 | 2.005 | 0.056^b^ |
| Eosinophils (%) | 5.14 | 2.64 | 0.49 |  | 6.72 | 4.64 | 0.93 |  | -1.76 | 5.68 | 1.14 | -4.1 | 0.58 | -1.549 | 0.134^b^ |
| Basophils (%) | 0 | 0 | 0 |  | 0 | 0 | 0 |  | - | - | - | - | - | - | ^-^ |
| Mean cell volume (fL) | 89.4 | 8.5 | 1.58 |  | 86.12 | 10.52 | 2.1 |  | 4.7 | 14.24 | 2.85 | -1.18 | 10.58 | 1.65 | 0.112^b^ |
| Mean corpuscular hemoglobin (pg) | 31.22 | 3.42 | 0.64 |  | 30.8 | 1.72 | 0.34 |  | 0.9 | 4.05 | 0.81 | -0.77 | 2.57 | 1.108 | 0.279^b^ |
| Mean corpuscular hemoglobin concentration (g/dL) | 34.86 | 1.18 | 0.22 |  | 34.7 | 1.26 | 0.25 |  | 0.13 | 1.86 | 0.37 | -0.64 | 0.9 | 0.35 | 0.73^b^ |

^a^Pair wise comparison of each parameter between baseline (day 0) and day 21 was performed using paired t-test.

^b^No significant difference was observed in most of the parameters between baseline (day 0) and day 21.
